# Observed Antibody Space: A diverse database of cleaned, annotated, and translated unpaired and paired antibody sequences

**DOI:** 10.1002/pro.4205

**Published:** 2021-10-29

**Authors:** Tobias H. Olsen, Fergus Boyles, Charlotte M. Deane

**Affiliations:** ^1^ Department of Statistics University of Oxford Oxford UK

**Keywords:** annotated antibody sequences, antibody database, antibody repertoire, antibody sequence, BCR‐seq, Observed Antibody Space (OAS)

## Abstract

The antibody repertoires of individuals and groups have been used to explore disease states, understand vaccine responses, and drive therapeutic development. The arrival of B‐cell receptor repertoire sequencing has enabled researchers to get a snapshot of these antibody repertoires, and as more data are generated, increasingly in‐depth studies are possible. However, most publicly available data only exist as raw FASTQ files, making the data hard to access, process, and compare. The Observed Antibody Space (OAS) database was created in 2018 to offer clean, annotated, and translated repertoire data. In this paper, we describe an update to OAS that has been driven by the increasing volume of data and the appearance of paired (VH/VL) sequence data. OAS is now accessible via a new web server, with standardized search parameters and a new sequence‐based search option. The new database provides both nucleotides and amino acids for every sequence, with additional sequence annotations to make the data Minimal Information about Adaptive Immune Receptor Repertoire compliant, and comments on potential problems with the sequence. OAS now contains 25 new studies, including severe acute respiratory syndrome coronavirus 2 data and paired sequencing data. The new database is accessible at http://opig.stats.ox.ac.uk/webapps/oas/, and all data are freely available for download.

## 1 INTRODUCTION

**FIGURE 1:**
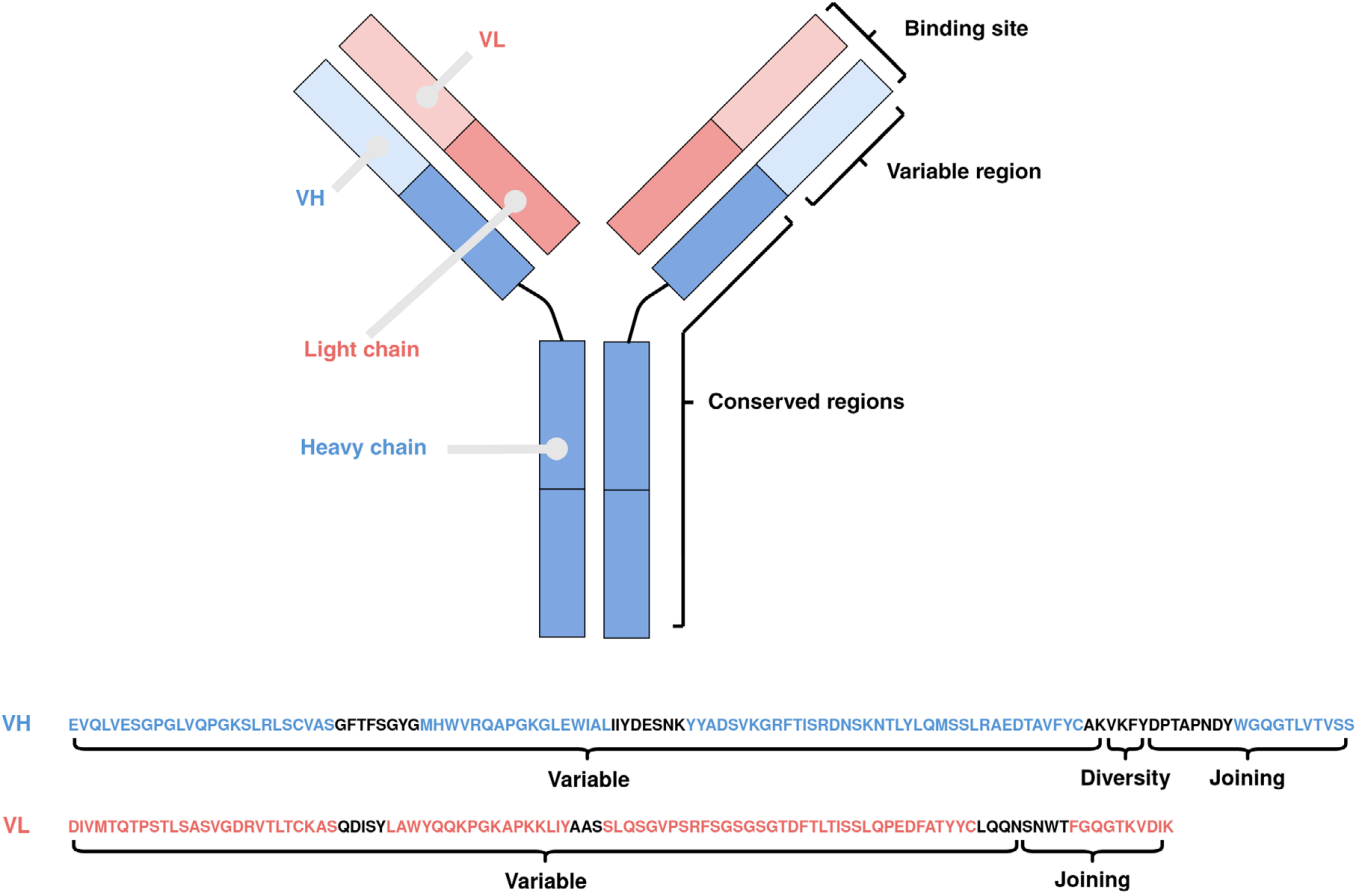
Overview of the structure of an antibody and the sequence of its variable regions. An antibody contains two heavy (blue) and two light (red) chains, with each chain separable into one or more conserved (C) and one variable (V) region. The paired heavy and light V regions, annotated as VH and VL respectively, contain the binding site

Antibodies are a central part of the immune system due to their ability to bind a wide range of disease molecules (antigens), either blocking their function or marking them for removal.^1^, ^2^ They consist of two large chains and two smaller chains, called heavy and light respectively, and each chain can be divided into one or more conserved (C) and one variable (V) region (Figure 1). In humans, the light chain comes from either the *κ* or *λ* loci, while the heavy chains come from a single locus. The C regions of the heavy chain can be of five different isotypes, IgA, IgD, IgE, IgG, and IgM, differing the biological properties and functional location of the antibody.^3^, ^4^, ^5^

Located at the tips of antibodies are the heavy chain variable region (VH) and the light chain variable region (VL), which paired together form the antigen binding site.^2^, ^4^ The V regions are encoded by multiple gene segments and are spliced together by a process known as V(D)J recombination, with the largest of these segments (variable segment) lending its name from the variable region.^6^ Further diversity is introduced by somatic hypermutation, especially in the three complementarity determining regions of each chain (shown in black in Figure 1).^3^, ^4^ Due to the high variability of the binding site, antibodies have the ability to bind with high affinity and specificity to almost any antigen and are the most important class of biotherapeutics.^1^, ^7^

The entire set of antibodies produced in an individual is called their antibody repertoire, also referred to as immunoglobulin (Ig) repertoire or B‐cell receptor (BCR) repertoire.^8^, ^9^ As the BCR repertoire represents the immunological condition of an individual,^10^ it can be used to investigate their immune states (effects from diseases or vaccination responses) and for finding and developing potential therapeutics.^7^, ^10^

However, the number of antibody sequences in a human antibody repertoire is estimated to be between 10^12^ and 10^15^ unique sequences, making it infeasible at present, to sequence every single antibody in the repertoire.^2^, ^11^ Moreover, the limited sequencing length of currently available high‐throughput sequencing platforms means the full antibody cannot be reliably sequenced.^9^ Antibody repertoire sequencing (BCR‐seq), therefore, focuses on sequencing the VH and VL regions, as these regions contain most of the variability and the binding site.^9^

Over recent years, BCR‐seq has been steadily improving.^8^ Growing from hundreds of thousands of unpaired sequences per sample,^12^, ^13^ to hundreds of millions of unpaired sequences.^14^, ^15^ Furthermore, with the introduction of single‐cell RNA sequencing techniques, it is now possible to sequence paired VH and VL.^16^, ^17^

The development of BCR‐seq techniques allows researchers to probe the diversity of the repertoire and the similarities and differences between individuals and different species.^9^ There are now tens of BCR‐seq datasets available containing millions of sequences from a variety of different hosts, diseases, treatments, and cell types.^10^ The use of data from multiple studies allows larger and more complete analyses to be carried out, but is hindered by nonstandard data formats. To counter this, the Adaptive Immune Receptor Repertoire (AIRR) Community proposed the Minimal Information about AIRR (MiAIRR) standard,^18^ which outlines a set of minimal required reported information. However, even with MiAIRR, a wide range of different processing pipelines still exist, complicating direct comparisons.^19^ Furthermore, the standard data release contains only the raw FASTQ files, therefore, to utilize the available data, it is often necessary to carry out extensive processing.^19^

As a response to this, in 2018, Kovaltsuk et al.^20^ created the Observed Antibody Space (OAS) database. OAS was a database of unpaired VH and VL antibody protein sequences, derived by identically processing 55 BCR‐seq datasets containing 600 million sequences.^20^ Related efforts include ImmuneAccess,^21^ pan immune repertoire database (PIRD)^22^ and rep‐seq dataset analysis platform with an integrated antibody database (RAPID),^23^ and the AIRR Data Commons^24^ (ADC), a network of geographically distributed AIRR compliant repositories accessible through a single API. These databases are a good source of annotated antibodies and include access to antibody visualization and analysis tools. ImmuneAccess holds a large set of annotated CDR3 sequences and RAPID identically processed human antibodies. PIRD and ADC contain large collections of annotated BCR‐seq, processed and deposited by independent researchers.

Since the first publication of OAS, an increased volume of data has been published as well as the appearance of paired (VH/VL) sequence data, leading to a need to expand and overhaul OAS.

In this paper, we present an updated and expanded OAS. The database now contains 1.5 billion unpaired sequences from 80 studies, including recent studies featuring severe acute respiratory syndrome coronaVirus 2 (SARS‐CoV‐2) data, and paired sequencing data from five studies. The new database also now provides the nucleotides for the VH and VL chains, in addition to amino acids. It also now contains additional sequence annotations, such as the antibodies junction sequence and whether it is a productive sequence, making the data MiAIRR‐compliant. Comments on potential problems (e.g., lack of conserved cysteines or unusual insertions and deletions) with the sequence have also been added. OAS is accessible via a new web server (http://opig.stats.ox.ac.uk/webapps/oas/), which provides standardized search parameters and a new option to search for sequences with the same V and J genes as a query sequence, allowing for a fast initial query of 1,000 antibody sequences similar to a given sequence of interest.

## 2 RESULTS

We have created an unpaired and paired OAS database using the approach given in Section 4. Unpaired OAS consists of the 55 BCR‐seq repertoires from the original OAS^20^ and, as of 30 September, 2021, 25 new studies. From the 80 studies, a total of 3,549,291,485 sequences (3,453,356,223 VH and 95,935,262 VL) and 1,535,831,565 unique sequences (1,499,142,547 VH and 36,689,018 VL) were retrieved. Heavy chains with all five isotypes are present in the database. The majority are IgM (1,019,175,618), IgG (287,174,563), and undetermined Bulk (167,366,413). B‐cell specific information is also included, with most of the sorted antibodies derived from Naive‐B‐Cells (125,521,501) or Plasma‐B‐Cells (40,126,155), and the bulk of the B‐cells extracted from leukopaks/peripheral blood (1,268,447,750) or the spleen (121,114,530).

Information about the individuals from which the BCR‐seq repertoires are taken was annotated where possible. The sequences can be mapped to 825 unique subjects and six different species, with the majority coming from humans (88%) and mice (11%). If given, age is annotated as precisely as possible, either as the exact age or as an age group. Available data include ages younger than 18 (253,056,094), between 18 and 70 (875,775,859) and over 70 (32,964,094). For any study where the same subject had a sample sequenced at different time points, the difference in time is annotated.

The data also covers a diverse set of immune states; 233,686,163 sequences are seen in individuals with 25 different diseases, including 61,730,169 and 47,853,000 sequences from SARS‐CoV‐2 and HIV infected patients, respectively. Twenty different vaccine combinations are present in unpaired OAS, with the majority of individuals vaccinated with HepB (52,111,613) or OVA (40,743,076).

Paired OAS contains 121,838 sequence pairs from five studies. Although less diverse, paired OAS already contains 11,388 pairs from SARS‐CoV‐2 infected patients and 17,913 pairs from Memory‐B‐Cells.

For every sequence in OAS, information about its nucleotide sequence, including the constant region if given, amino acid sequence, chain, isotype, and annotated germline is specified. To make OAS MiAIRR compliant, information required by AIRR's rearrangement schema, such as whether a sequence is productive, is also included. The IMGT numberings and an ANARCI status have been added to highlight any deletions, insertions, missing conserved cysteines, or truncated ends.

**FIGURE 2:**
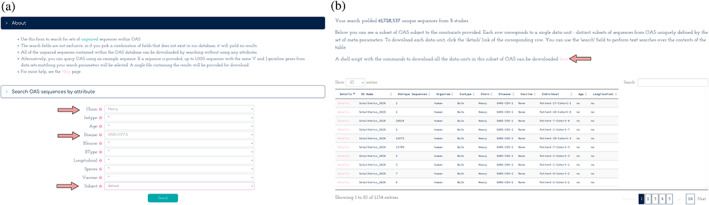
Downloading from OAS. (a) The sequence search tab for unpaired sequences, with the search options filled for heavy chain sequences from SARS‐CoV‐2 infected patients (shown with red arrows). (b) The search result, with each data unit matching the search and a downloadable link containing the links for the relevant data units (with a red arrow)

To allow for a simple extraction of the data present in OAS, or subsets of it, we have created the OAS website http://opig.stats.ox.ac.uk/webapps/oas/. In addition to the ability to download all of OAS, the data can be queried by all meta labels (e.g., study, species, chain, and disease). As an example, to compare heavy chain sequences from different patients infected with SARS‐CoV‐2, one could go to the unpaired data and select Chain: Heavy, Disease: SARS‐CoV‐2, and Subject: Defined. The website will then provide links to download files containing 61 million unique sequences from five studies and 130 different SARS‐CoV‐2 patients (Figure 2).

We have also added a sequence search option, allowing a user to select sequences in OAS that are similar to a query sequence. The search will return a sample of 1,000 sequences from the database with the same V and J germline genes as the query sequence and can be combined with the meta label search to obtain, for example, a sample of 1,000 mouse heavy chain sequences with the same V and J gene as a given query.

## 3 DISCUSSION

Antibody repertoires have become crucial datasets for examining the immune response and the development of antibodies as therapeutics. Although large BCR‐seq datasets from a variety of studies exist, data processing is often inconsistent between datasets, or the data are only publicly available as raw FASTQ files. This prompted us to create OAS, a resource of BCR‐seq datasets all processed in a standard manner. In this paper, we present an updated version of OAS featuring an improved processing pipeline, additional sequence information, an increased amount of unpaired data, and the inclusion of paired data.

In the processing pipeline, germline is now annotated using nucleotides, instead of amino acids, allowing for better annotations. Moreover, additional information, for example nucleotides and ANARCI status, and new data, for example, SARS‐CoV‐2 infected patients, has been added. Most importantly, we also introduce paired OAS. Paired data will allow investigations of the whole binding site, instead of solely the heavy or light chain.

To make OAS more findable, accessible, interoperable, and reusable (FAIR),^25^ OAS has been adapted to be compliant with MiAIRR. The first four sets of MiAIRR are associated with the creation of FASTQ files. As we cannot control the creation of the FASTQ files, we instead include the source in the metadata so a sequence can always be linked back to its original study and run. The fifth MiAIRR set encompasses data processing. Here, all sequences are processed as elaborated in Section 4 with the software version given for each tool used. Finally, the sixth MiAIRR set is followed by including all the required elements in MiAIRR's rearrangement schema. By following this schema, OAS can be merged with data from other MiAIRR compliant sources, such as the ADC, and interoperate with the same tools. However, as data from the ADC are not processed using a single standardized processing pipeline, and those pipelines are also potentially different from OAS, this presents a challenge when reusing studies from different databases for direct comparisons, and for creating truly FAIR repertoire data.

To further improve the accessibility, OAS is freely available at http://opig.stats.ox.ac.uk/webapps/oas/, where the newest processed unpaired and paired data will be added to OAS over time. Subsets of interest can be queried and downloaded based on all meta labels from the website. In addition, users can perform a search for sequences with the same V and J gene allowing for a fast initial exploration of a sequence of interest.

This updated and expanded version of OAS is a versatile and valuable resource for a wide range of antibody research areas, including investigation of immune states, by enabling comparison of antibody repertoire data between studies, and therapeutic antibody design, by giving access to more than 1.5 billion cleaned, annotated, and translated antibody sequences, paired VH/VL, and facilitate a fast exploration of similar antibody sequences.

## 4 MATERIALS AND METHODS

Accession numbers of publicly available BCR‐seq datasets were extracted from the literature, and NCBI's SRA Run Selector^26^ was used to download the metadata of each run within a study. The metadata was then processed into a standardized format, which contains the run accession number, the author and associated DOI, subject identifier, age of subject, species, B‐cell source, B‐cell type, vaccinations, diseases, and any longitudinal information.

The FASTQ files of each run were then downloaded using SRA‐Tools fastq‐dump 2.9.1.^27^ For runs sequenced using a paired‐end library layout, FLASH 1.2.11^28^ was used to merge the FASTQ files into a single file. Unpaired sequences were then converted from a FASTQ file into a FASTA file using fastq_to_fasta from the FASTX‐Toolkit 0.0.14.^29^ Paired sequences were converted to a FASTA file using 10× Genomics Cell Ranger 6.0.2.^30^ The barcode in the resulting FASTA file was then used to map heavy and light chains that form a pair. The unpaired and paired datasets then follow the same processing steps.

The nucleotide‐containing FASTA file was aligned to germline and translated using IgBLASTn 1.17.1.^31^ For IgBLASTn, the VDJ germline databases for human, mouse, rat, rabbit, and monkey were created using ImMunoGeneTics (IMGT) germline sequences derived from IMGT.^32^ For camel sequences, the nearest relative alpaca was used. Sequences deemed unproductive by IgBLASTn or with no resulting aligned sequence were removed. To determine the isotype of heavy chain sequences, the first 21 nucleotides of the constant heavy region 1 (CH1), obtained from IgBLASTn, were aligned to a set of IMGT‐derived constant region germline with annotated isotypes. The alignment was done using the same conservative alignment approach as used by Kovaltsuk et al.,^20^ and sequences with no high‐confidence alignments or with missing CH1s had their isotype annotated as Bulk.

For each sequence, the IMGT numbering scheme was added using antibody numbering and antigen receptor classIfication (ANARCI) April 23, 2020.^33^ Any sequence that ANARCI could not process was removed. This step predominantly removes sequences that contain a stop codon. An ANARCI status highlighting potential problems for each sequence is retained in the database. This status contains comments regarding unusual residues, lack of conserved cysteines, deletions and insertions outside of the CDRs, truncation of frameworks 1 or 4, and if the CDR3 is longer than 37 residues. Finally, sequences were grouped into units sharing the same metadata, the same chain (e.g., heavy, light, or paired), and isotype.

## AUTHOR CONTRIBUTIONS

**Tobias Hegelund Olsen:** Conceptualization (equal); data curation (equal); formal analysis (equal); funding acquisition (equal); methodology (equal); software (equal); validation (equal); writing – original draft (equal). **Fergus Boyles:** Conceptualization (equal); data curation (equal); funding acquisition (equal); software (equal); writing – review and editing (equal). **Charlotte Deane:** Conceptualization (equal); funding acquisition (equal); project administration (equal); resources (equal); supervision (equal); writing – review and editing (equal).

## CONFLICT OF INTEREST

The authors declare no conflicts of interest.
